# Gαo Is Required for L-Canavanine Detection in *Drosophila*

**DOI:** 10.1371/journal.pone.0063484

**Published:** 2013-05-06

**Authors:** Isabelle Devambez, Moutaz Ali Agha, Christian Mitri, Joël Bockaert, Marie-Laure Parmentier, Frédéric Marion-Poll, Yves Grau, Laurent Soustelle

**Affiliations:** 1 CNRS, UMR 5203, Institut de Génomique Fonctionnelle, Montpellier, France; 2 INSERM, U661, Montpellier, France; 3 Universités de Montpellier 1 & 2, UMR 5203, Montpellier, France; 4 INRA, UMR 1272, Physiologie de l'Insecte, Versailles, France; 5 Université Pierre et Marie Curie, UMR 1272 Physiologie de l'Insecte, Versailles, France; 6 AgroParisTech, Département Sciences de la Vie et Santé, Paris, France; 7 Institut Pasteur, URA3012 CNRS, Unité Génétique et Génomique des Insectes Vecteurs, Paris; German Institute for Human Nutrition, Germany

## Abstract

Taste is an essential sense for the survival of most organisms. In insects, taste is particularly important as it allows to detect and avoid ingesting many plant toxins, such as L-canavanine. We previously showed that L-canavanine is toxic for *Drosophila melanogaster* and that flies are able to detect this toxin in the food. L-canavanine is a ligand of DmXR, a variant G-protein coupled receptor (GPCR) belonging to the metabotropic glutamate receptor subfamily that is expressed in bitter-sensitive taste neurons of *Drosophila*. To transduce the signal intracellularly, GPCR activate heterotrimeric G proteins constituted of α, β and γ subunits. The aim of this study was to identify which Gα protein was required for L-canavanine detection in *Drosophila*. By using a pharmacological approach, we first demonstrated that DmXR has the best coupling with Gα_o_ protein subtype. Then, by using genetic, behavioral assays and electrophysiology, we found that Gαo47A is required in bitter-sensitive taste neurons for L-canavanine sensitivity. In conclusion, our study revealed that Gαo47A plays a crucial role in L-canavanine detection.

## Introduction

Taste is an important chemosensory cue, which is crucial for the survival of any organisms as it prevents the ingestion of toxic compounds. Toxins often have a bitter taste, explaining why the activation of bitter-sensitive taste neurons is generally associated with a rejection behavior. This reaction to bitter molecules is found in vertebrates but also in the fruit fly *Drosophila*, which react similarly to human for various tastants [1].

As a defense mechanism against predators, plants have developed toxins and antifeedants such as L-canavanine. The toxicity of L-canavanine is due to its structural similarities with L-arginine, leading to its incorporation into *de novo* synthesized proteins, making them not functional [2]. We previously showed that forced ingestion of L-canavanine is deleterious to *Drosophila melanogaster* and that this organism has the capacity to detect the presence of L-canavanine into the food, preventing its ingestion [3]. Thus, L-canavanine acts as a repellent molecule for fruit flies.

*Drosophila* taste neurons (also called gustatory receptor neurons, GRNs) are found in sensilla that are localized in the proboscis, legs, wings as well as the ovipositor [4]. Each sensillum houses two to four GRNs, which are dedicated to different taste modalities. Indeed, *Drosophila* gustatory system is able to detect sugars, bitter/toxic compounds, salts and water [4]. Recent studies have also shown that the *Drosophila* gustatory system is involved in pheromone detection and plays a role in courtship [5], [6].

The first family of taste receptors identified in *Drosophila melanogaster* were members of the Gustatory Receptors (GRs) family that include 60 genes predicted to encode 68 proteins generated by alternative splicing. Most of them are expressed in bitter-sensitive GRNs [7]. In addition, most if not all bitter-sensitive GRNs express GR66a, which was originally described as a caffeine receptor [8]. Caffeine is repellent for *Drosophila* and its detection not only requires GR66a but also, at least, GR33a and GR93a as the mutation of one of these three GRs impaired caffeine detection [9]. Also, Lee and collaborators found that the detection of the synthetic repellent compound DEET required GR32a, GR33a, and GR66a and suggested that GRs may act in a heteromultimeric complex [10]. In addition, it was suggested that GR33a is an indispensable co-receptor for bitter compounds as GR33a mutant flies are impaired for the perception of most of them [9]. A similar situation was found for the detection of most sugars, where it was shown that GR64f is a co-receptor of GR5a and GR64a [11]. Because GRs are seven transmembrane proteins, it was originally thought that they were G-protein coupled receptors (GPCRs) [12], [13]. However, GRs, like members of the related olfactory receptor (OR) family, have an inverted topology compared to GPCRs [14], [15], [16]. Recent studies have highlighted the repertoire diversity of taste receptors in *Drosophila*. Indeed, members of the degenerin/epithelial sodium/pickpocket (DEG/EnaC/ppk) channel family are involved in water and salt taste detection [17], [18], [19] and TRPA1, a member of the Transient Receptor Potential (TRP) channel family, detects reactive electrophiles [20], such as allyl isothiocyanate, which gives a pungent taste to mustard and wasabi.

We have previously published that L-canavanine detection and associated behaviors relie on a GPCR called DmX [3]. The DmX receptor belongs to the metabotropic glutamate receptor (mGluR) family but it is not activated by glutamate due to conserved modifications within its ligand binding pocket [21]. We also found that L-canavanine binds and activates DmXR in HEK transfected cells [3]. However, a recent report has also shown that GR66a and GR8a, two members of the GR family, were involved in L-canavanine detection [22].

Canonical GPCR signaling relies on an intracellular heterotrimer of G proteins constituted of one Gα, one Gβ and one Gγ subunit. In its inactive state, the Gα subunit is bound to GDP. Upon GPCR activation, GDP is replaced by GTP and subsequently GTP-bound Gα and Gβ/γ subunits dissociate to activate downstream effectors [23]. Classically, mammalian Gα proteins are divided into four subfamilies based on sequence similarities: Gα_s_, Gα_i/o_, Gα_q/11_ and Gα_12/13_ [24]. The Gα_s_ and Gα_i/o_ subfamilies were named for their ability to stimulate and inhibit adenylyl cyclase isoforms, respectively. The Gα_q/11_ subfamily is linked to the stimulation of phospholipase Cβ while the Gα_12/13_ subfamily activates the small G protein Rho pathways [24].

Here to better understand the signaling pathway involved in L-canavanine detection in bitter-sensitive taste neurons, we focused on G proteins, asking if any Gα is required for L-canavanine sensitivity. We first used a pharmacological approach to determine which Gα protein has the best coupling to DmXR and found that DmXR can transduce the signal via Gα_o_ subtype in HEK transfected cells. Then, we performed genetic and behavioral experiments and found that Gαo47A, the only Gα_o_ member in the *Drosophila melanogaster* genome, is required in bitter-sensitive taste neurons for L-canavanine detection. Finally, by using an electrophysiological approach, we confirmed that blocking Gαo47A function led to a very strong reduction in L-canavanine sensitivity and has no other impact on the bitter taste neurons, as caffeine detection was normal.

Altogether, our data showed that Gαo47A is required for L-canavanine detection in bitter-sensitive taste neurons of *Drosophila*.

## Materials and Methods

### Cell culture, transfection and inositol phosphate (IP) assay

HEK 293 cells were cultured as described in [25] and transiently transfected by electroporation with either 14 µg of carrier DNA (pRK), plasmid DNA containing HA-DmXR wild-type, plasmid DNA containing Gα protein (2 µg) (into pcDNA3.1, Invitrogen). Several Gα proteins were used, including wild type (Gα_15_, Gα_16_, Gα_q_) or chimeric (Gα_qo5_, Gα_qi9_, Gα_qZ5_) proteins [26]. All these wild type and chimeric Gα proteins are known to activate phospholipase C [26]. Determination of inositol phosphate (IP) accumulation in transfected cells was performed after labeling the cells overnight with ^[3H]^myoinositol (23.4 Ci/mol) as described previously [27]. The stimulation was conducted for 30 min in a medium containing 10 mM LiCl and 10 mM L-canavanine. The basal IP formation was determined after 30-min incubation in the presence of 10 mM LiCl. Results are expressed as the amount of IP produced divided by the radioactivity present in the membranes. L-canavanine was purchased from Sigma (#c1625).

### Fly stocks

CantonS flies were used as wild-type and w^1118^ flies were used as a control for electrophysiological experiments. Gr66a-Gal4 line was a gift from H. Amrein (Texas A&M Health Science Center, College Station). UAS-RNAiGαi65A (stock 28150) and UAS-RNAiGαo47A (stock 19124) lines were obtained at the Vienna *Drosophila* RNAi Center (VDRC). UAS-Go^GDP^ carried a mutant form of Gα_o_ (G203T mutation), which mimicked the GDP-bound state of Gα_o_ protein [28]. This line was a gift from A. Tomlinson (Columbia University). The UAS-PTX line was a gift from G. Roman (University of Houston) [29].

### PER/PPR assay

The proboscis extension reflex (PER) and the premature proboscis retraction (PPR) were examined as described in [3]. Briefly, adult flies were maintained on fresh medium and then starved on water-saturated cotton for 20 h. Flies were then immobilized by chilling them on ice and mounted ventral-side-up using myristic acid. Flies were allowed to recover for two hours in humid conditions. Before the assay, flies were satiated with water until no proboscis extension was elicited by water stimulation. Each fly was tested during 5 s by touching only the leg tarsi with either a 100 mM sucrose solution or 100 mM sucrose+40 mM L-canavanine solution. Six to eight batches of 40–60 flies were tested for each solution and each genotype. The occurrence of PER and PPR was determined during the assay. The percentage of PPR represents the number of flies that showed the PPR phenotype divided by the number of flies that have shown a PER. Unpaired Student t-tests were used to check for significant differences between the indicated pairs of data.

### Two-choice feeding test

For each trial, between 80 to 100 adult flies (3- to 5-days old) were starved on water-saturated cotton for 24 h. Flies were then placed on a 60-well microtiter plate (#56243, Dutschern France) at 25°C during two hours in the dark. Wells contained 1% agarose with 0.15 mg/ml erioglaucine dye (blue) or 0.2 mg/ml sulforhodamine B dye (red) in the alternating wells. The sucrose concentrations were 5 and 1 mM in the blue and red solutions, respectively. After 2 h on the plates, the flies were frozen and the numbers of flies that were blue (NB), red (NR), or purple (NP) were determined on the basis of the colors of their abdomen. The preference index (PI) values for the blue solution were calculated according to the following equation: (NB+0.5NP)/(NB+NP+NR). A PI value of 1 or 0 indicates a complete preference or aversion, respectively. A PI value of 0.5 indicates no preference/aversion. In all the tests shown, the L-canavanine was added to the blue solution. Four independent trials were carried out for each condition. Unpaired Student t-tests were used to check for significant differences between the indicated pairs of data.

### Electrophysiological recordings

For electrophysiological recordings, 4 days old flies were briefly numbed in ice and then restrained on their side on putty (UHU Patafix®), using fine strips of semi-transparent tape. A silver wire connected to the electrical ground was maintained close to their abdomen and a drop of electrocardiogram gel (Redux Gel, Parker Laboratories, Fairfields NJ, USA) was then deposited over it, thus providing an electrical reference and ensuring a minimal stress to the insect. The preparation was then left to rest about 30 min to 1 h before recordings occurred. The preparation was brought under a microscope (Leica MZ16), and properly oriented so that the S6 sensillum on the proboscis was accessible to stimulation (see map from [30]). As for stimulation, we used borosilicate glass capillaries (tip size about 10 µm; Harvard Apparatus LTD, EdenBridge, UK), filled with the stimulus solution and 1 mM KCl, which served as an electrolyte. This electrode was connected to a taste amplifier (TastePROBE DT-02, [31]), which triggered upon contact a 2 seconds recording bout with a 16 bits data acquisition board (DT9803, Data Translation, USA) sampling data at 10 kHz, under the control of a custom program (dbWave; [32]). Data were further amplified (×500) and filtered (10–2800 Hz) with a CyberAmp 320 amplifier (Axon Instruments, USA). The number of spikes occurring during each recording was detected using dbWave and exported to a spreadsheet for further analysis. Unpaired Student t-tests were used to check for significant differences between the indicated pairs of data.

## Results

### The G-protein coupled receptor DmX is coupled to Gα_i/o_ protein subtype *in vitro*

**Figure 1:**
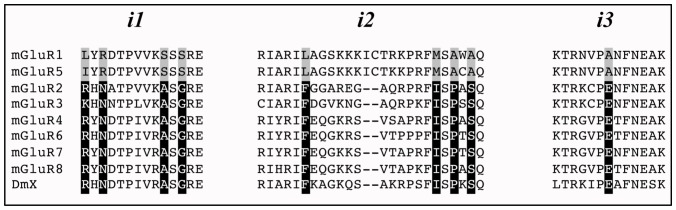
Sequence alignment of the intracellular loops of mGluRs and DmXR. *i1*, *i2*, and *i3* correspond to the first, second, and third intracellular loops of mGluRs and DmXR, respectively. Residues conserved in mGluRs coupled to phospholipase C (mGluR1 and 5) are boxed in grey, and the corresponding residues in most adenylyl cyclase coupled mGluRs (mGluR2, 3, 4, 6, 7 and 8) and DmXR are boxed in black, respectively.

DmXR belongs to the metabotropic glutamate receptors (mGluRs) subfamily, which includes eight members in vertebrates. mGluR1-5 are positively coupled to phospholipaseC (PLC) via Gα_q_, while mGluR2,3,4,6,7 and 8 are negatively coupled to adenylyl cyclase via Gα protein of i/o subtype [33]. The intracellular domains of mGluRs have been extensively studied and are responsible for the specificity of coupling to specific G-proteins, especially the second intracellular loop [34], [35]. Hence, all Gα_i/o_ coupled mGluRs share identical residues at different positions of the intracellular loops, and these residues are different in mGluR1 and 5 (Fig. 1). To get a hint on the G-protein-coupling specificity of DmXR, we first analyzed its intracellular loop sequences and found that DmXR share the conserved residues of Gα_i/o_-coupled mGluRs instead of those of mGluR1 and 5 (Fig. 1). Thus, DmXR may be coupled to Gα_i_ or Gαo, or both.

The ability of individual Gα protein to discriminate specific GPCRs is linked to the presence of specific residues localized within the C-terminal region of the Gα subunits [26]. Taking advantage of this observation, chimeric Gα proteins have been made by replacing the 5 to 9 C-terminal residues of Gα_q_ protein by the corresponding residues of Gα_i/o_ or Gα_z_ proteins (the latter being a divergent member of the Gα_i/o_ family). These proteins are denoted Gα_qi9_, Gα_qo5_ and Gα_qz5_ respectively. Importantly, the coupling specificity of these chimeric Gα proteins towards GPCRs is conserved [26], *i.e.* Gα_qi9_ is activated by Gα_i_ coupled receptors. Note that these chimeric G-proteins activate PLC, like Gα_q_, instead of inhibiting adenylyl cyclase [26]. Hence, these chimeric proteins, as well as other wild-type Gα proteins that activate PLC (Gα_15_, Gα_16_ and Gα_q_) allows to characterize the G-protein coupling profile of DmXR by using a single *in vitro* assay: the measure of ligand-induced inositol phosphate (IP) production.

**Figure 2:**
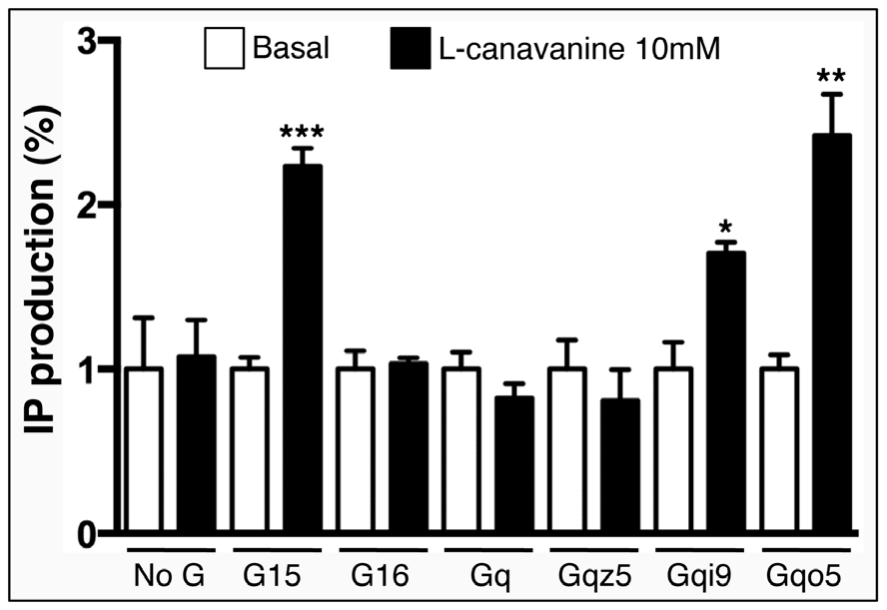
The GPCR DmX has the best coupling with Gαo protein subtype in HEK transfected cells. L-canavanine induced-inositol phosphate (IP) production was measured from HEK cells co-expressing the DmX receptor and the indicated Gα protein. As a control, we used HEK cells transfected with DmXR expression vector alone (called ‘No G’). Basal and 10 mM L-canavanine were used for all stimulations, indicated by white and black bars, respectively. IP stimulation was calculated relatively to IP production in basal conditions. HEK cells co-expressing DmXR and Gα_15_, Gα_qi9_ or Gα_qo5_ produced IP after L-canavanine stimulation, indicating that these Gα proteins can efficiently couple to DmXR, the best coupling being observed with Gα_qo5_. No such effect was observed with HEK cells co-expressing DmXR and Gα_16_, Gα_q_ or Gα_qz5_. Experiments done with Gα_15_ could be considered as a positive control because Gα_15_ protein is known to couple with most GPCRs. Data are means +/− SEM from triplicate experiments. IP production was compared with basal activity using Unpaired Student's *t* test (* p<0.05, ** p<0.01, *** p<0.001).

Human embryonic kidney (HEK) cells were co-transfected with expression vectors carrying DmXR without or with one Gα protein subtype, including wild type (Gα_15_, Gα_16_ and Gα_q_) or chimeric (Gα_qi9_, Gα_qo5_ and Gα_qz5_) proteins. We then measured the IP production in presence or absence of L-canavanine, the known ligand of DmXR [3]. Data shown in Fig. 2 indicate that the strongest L-canavanine-induced DmXR activation was found when HEK cells co-expressed Gα_qo5_. A weakest, but statistically significant, IP production was observed with HEK cells co-expressing DmXR and Gαqi9 (Fig. 2). As expected, we detected L-canavanine-induced DmXR activation by using the Gα_15_ protein, which is known to couple to most types of GPCRs [36], [37]. In contrast, no L-canavanine-induced DmXR activation was observed when HEK cells were co-transfected with Gα_16_, Gα_q_ or Gα_qz5_ (Fig. 2), indicating that DmXR was not coupled to such types of Gα proteins, at least in HEK cells. Thus, we conclude that DmXR is a GPCR that couples to Gα_i/o_ proteins.

### Gαo47A, but not Gαi65a, is required in bitter-sensitive neurons for L-canavanine-induced premature proboscis retraction

**Figure 3:**
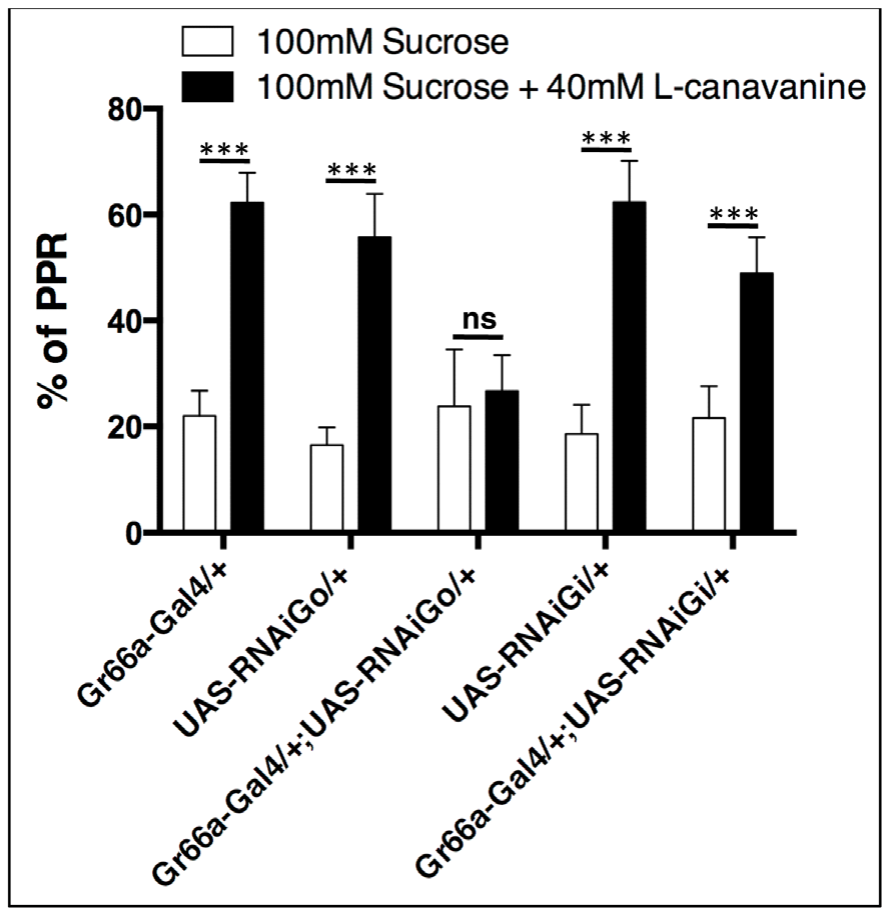
RNAi knockdown of Gαo47A in bitter-sensitive taste neurons impairs L-canavanine-induced premature proboscis retraction. L-canavanine-induced premature proboscis retraction (PPR) was analyzed with 100 mM sucrose solution (white bars) and a solution containing 100 mM sucrose+40 mM L-canavanine (black bars). For all genotypes, the percentage of PPR is very low when tarsi are stimulated with the sucrose solution. Gr66a-Gal4/+, UAS-RNAiGαo47A/+ (UAS-RNAiGo/+) and UAS-RNAiGαi65A/+ (UAS-RNAiGi/+) control flies as well as Gr66a-Gal4/+;UAS-RNAiGαi65A/+ (Gr66a-Gal4/+;UAS-RNAiGi/+) flies prematurely retract their proboscis when tarsi are in contact with a L-canavanine containing sucrose solution. On the contrary, the percentage of Gr66a-Gal4/+;UAS-RNAiGαo47A/+ (Gr66a-Gal4/+;UAS-RNAiGo/+) flies that prematurely retracted their proboscis is very low, indicating that these flies maintained their proboscis extended due to L-canavanine detection defects. Error bars indicate SEM. Asterisks indicate significant differences by Unpaired Student's *t* test (ns: not significant, *** p<0.001).

In the *Drosophila melanogaster* genome, two genes encoding Gα_i/o_ subtypes of G proteins are present: Gαi65A (CG10060) and Gαo47A (CG2204). In order to determine which Gα protein is required for L-canavanine detection *in vivo*, we used flies expressing specific RNAi against each of these two G proteins, specifically in bitter-sensitive taste neurons and performed behavioral analyses. One paradigm to study taste in flies is the proboscis extension reflex (PER) assay [38]. During this test, the stimulation of leg tarsi with a sucrose solution induces an extension of the proboscis, which is maintained several seconds. When a deterrent compound is added to a sucrose solution, the reflex is blocked and flies do not extend their proboscis. This inhibitory effect on sucrose-induced proboscis extension reflex was observed for most deterrent compounds such as caffeine, strychnine and quinine but not for L-canavanine [3], [38]. Indeed, we previously found that the stimulation of leg tarsi with a L-canavanine and sucrose mixed solution induced a premature proboscis retraction (PPR), *i.e.* the flies extended their proboscis but retracted it almost immediately [3]. By using the Gr66a-Gal4 driver, which targets all bitter-sensitive taste neurons, we expressed RNAi construct against Gαi65A or Gαo47A and analyzed PPR phenotypes in presence or not of L-canavanine. Data shown in Fig. 3 indicate that all genotypes tested had a very low percentage of PPR when a sucrose solution was used for leg tarsi stimulation, indicating that flies detected sucrose correctly and maintained their proboscis extended. In contrast, Gr66a-Gal4/+, UAS-RNAiGαo47A/+, UAS-RNAiGαi65A/+ control flies and Gr66a-Gal4/+;UAS-RNAiGαi65A/+ flies presented a high percentage of PPR when a L-canavanine and sucrose mixed solution was used (Fig. 3). This revealed that these flies detected L-canavanine and retracted prematurely their proboscis, excluding a role of Gαi65A in the signaling pathway linked with L-canavanine detection. On the contrary, a similar low percentage of PPR was obtained with the sucrose and the L-canavanine/sucrose mixed solution on Gr66a-Gal4/+;UAS-RNAiGαo47A/+ flies. This experiment demonstrates that the down-regulation of Gαo47A in bitter-sensitive taste neurons impaired L-canavanine sensitivity (Fig. 3). These data strongly suggest that Gαo47A, but not Gαi65A, plays a role in L-canavanine detection *in vivo*.

### L-canavanine detection is impaired in flies expressing Gαo47A RNAi or a dominant negative Gαo (Gαo^GDP^) in bitter-sensitive taste neurons

**Figure 4:**
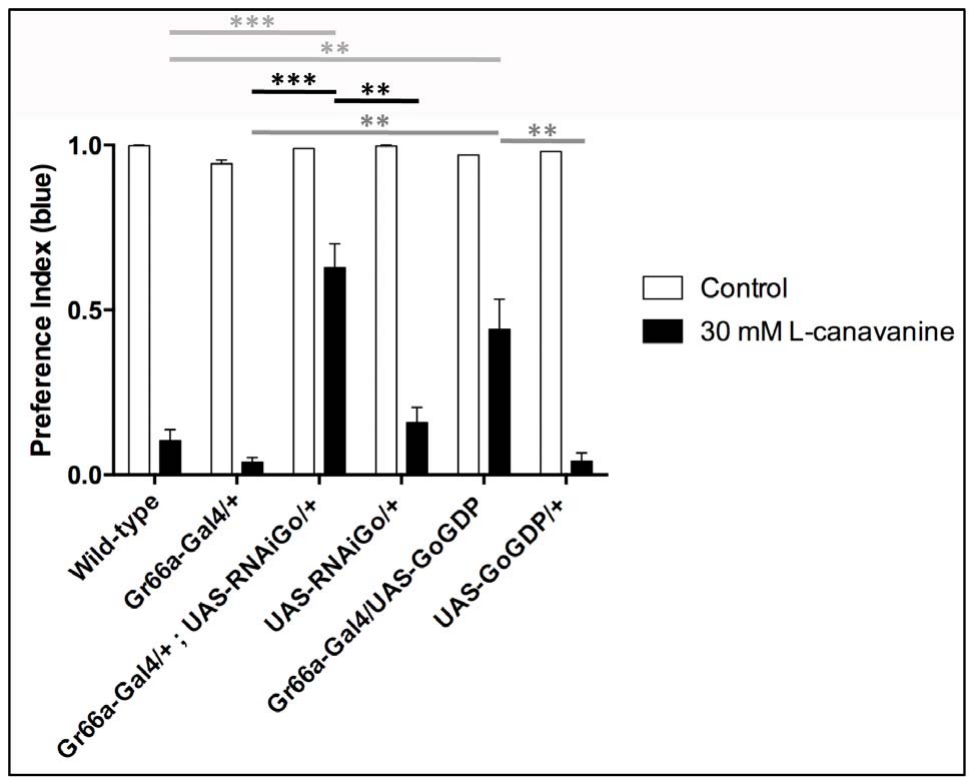
L-canavanine aversion is reduced when bitter-sensitive taste neurons express a RNAi construct against Gαo47A or a dominant negative form of Gαo47A. Two-choice feeding test experiments showing preference index (PI) for the blue solution of different genotypes. Control (white bars) and 30 mM L-canavanine (black bars) indicate that no drug or 30 mM L-canavanine was added to the blue solution, respectively. A complete preference or aversion is indicated by a PI value of 1 or 0, respectively. The down regulation of Gαo47A by RNA interference (Gr66a-Gal4/+;UAS-RNAiGo/+) and the inhibition of Gαo47A by using a dominant negative construct (Gr66a-Gal4/UAS-Go^GDP^) reduced the aversion to L-canavanine compared to controls (wild-type, Gr66a-Gal4/+, UAS-RNAiGo/+ and UAS-Go^GDP^/+). Note that all genotypes did not show any defect for sugar detection. Error bars indicate SEM. Asterisks indicate significant differences by Unpaired Student's *t* test (** p<0.01, *** p<0.001).

In order to confirm these data, we used another behavioral assay: the two choice feeding test, which measures the consumption of sucrose solutions colored by two food dyes of different colors (blue/red) offered simultaneously to flies. In this test, the blue solution contained more sucrose (5 mM) compared to the red one (1 mM), inducing an attraction of wild-type flies towards the blue solution as shown in Fig. 4 (wild-type in white bar). When L-canavanine (30 mM) was added to the blue sucrose solution, wild-type flies detected it and avoided eating the blue solution (Fig. 4, wild-type in black bar), consistently with the repulsive effect of L-canavanine. By using this test, we found that RNAi knock-down of Gαo47A in bitter-sensitive taste neurons impaired L-canavanine detection but had not effect on sucrose attraction (Fig. 4). Similar results were obtained with a Gαo mutant construct (Fig. 4), known to mimics the GDP bound Gαo (Gαo^GDP^) and which acts as a dominant negative of the Gαo^GTP^ form [28]. Note that the effect was stronger by using the Gαo47A RNAi than the Gαo^GDP^ construct (Fig. 4), likely because the RNAi was more efficient to block Gαo47A function. The same experiments were performed with flies expressing a RNAi construct against Gαi65A specifically in bitter-sensitive taste neurons. As shown in the Fig. S1.A, Gαi65A knock-down had no impact on L-canavanine detection, confirming the data obtained on PPR analysis. Altogether, these data indicate that L-canavanine detection requires the presence of Gαo47A, but not Gαi65A in bitter-sensitive taste neurons.

### Pertussis toxin inhibition of Gαo47A strongly reduced L-canavanine aversion

**Figure 5:**
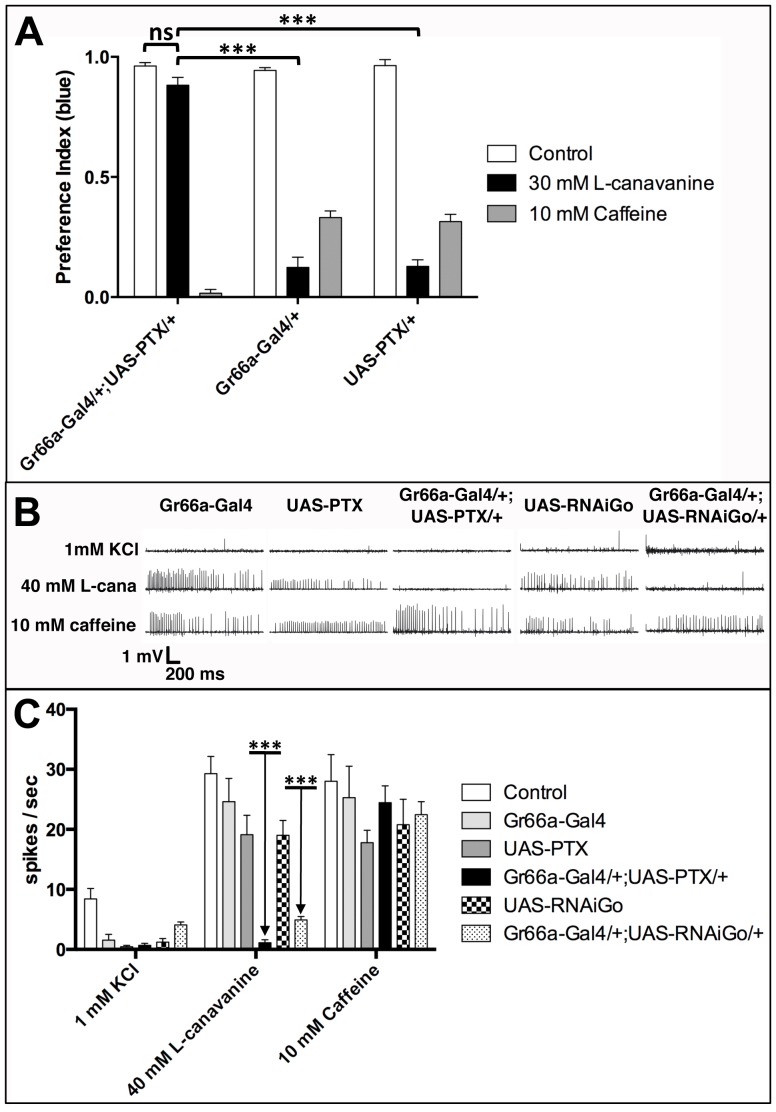
PTX inhibition of Gαo47A in bitter-sensitive taste neurons highly reduces L-canavanine aversion and L-canavanine-induced nerve firings, but has no effect on caffeine aversion. **A)** Two-choice feeding test experiments showing preference index for the blue solution of flies with different genotypes. Control indicates that no drug was added to the blue medium (white bars). Data obtained by using 30 mM L-canavanine in the blue medium are shown in black bars. The expression of a selective toxin (pertussis toxin, PTX) for Gαo47A in Gr66a-positive taste neurons (Gr66a-Gal4/+;UAS-PTX/+) highly reduces the aversion to L-canavanine compared to controls (Gr66a-Gal4/+ and UAS-PTX/+). Gr66a-Gal4/+;UAS-PTX/+ did not distinguish the control and the L-canavanine containing solutions (ns, p = 0.0526). Note that Gr66a-Gal4/+;UAS-PTX/+ flies are more sensitive to caffeine (grey bar) than the Gr66a-Gal4/+ and UAS-PTX/+ control lines (p<0.001). Error bars indicate SEM. Asterisks indicate significant differences by Unpaired Student's *t* test (ns: not significant, *** p<0.001). **B–C)** Electrophysiological recordings were performed from s6 sensilla on the proboscis of flies with different genotypes. The electrical activity of the taste neurons was recorded by capping taste sensillum with an electrode containing 1 mM KCl as an electrolyte and the stimulus (40 mM L-canavanine or 10 mM caffeine). **B)** Sample responses for 1 mM KCl, 40 mM L-canavanine (mentioned as L-cana) and 10 mM caffeine on Gr66a-Gal4,UAS-PTX, Gr66a-Gal4/+;UAS-PTX/+, UAS-RNAiGαo47A and Gr66a-Gal4/+;UAS-RNAiGαo47A/+ flies. **C)** Compared to control (white bars) and parental lines (light grey, dark grey and squared bars), Gr66a-Gal4/+;UAS-PTX/+ (black bars) and Gr66a-Gal4/+;UAS-RNAiGαo47A/+ (dotted bars) did not respond to 40 mM L-canavanine. Note that the response to 10 mM caffeine is not altered for all genotypes. The response was evaluated by counting the number of spikes elicited during the first second of the stimulation. N = 7–10 for each condition. Error bars indicate SEM. Asterisks indicate significant differences by Unpaired Student's *t* test (*** p<0.001).

To further demonstrate that Gαo47A is involved in L-canavanine detection, we took advantage of a transgenic line carrying the gene encoding for Pertussis toxin (PTX) under the control of UAS sequence. In vertebrates, PTX is known to specifically block the function of Gα_i_ and Gα_o_ proteins by catalyzing the ADP-ribosylation of these G proteins at a conserved C-terminal cysteine [39]. However, in *Drosophila melanogaster*, it is well established that PTX inhibits only Gα_o_, as the Gα_i_ protein does not contain this cysteine [40]. We crossed the Gr66a-Gal4 line with the UAS-PTX line and analyzed the behavior of the progeny (Gr66a-Gal4/+;UAS-PTX/+) by using two-choice feeding test experiments. As shown on Fig. 5A, the progeny of the parental lines crossed with wild-type flies had a normal taste behavior, *i.e.* Gr66a-Gal4/+ and UAS-PTX/+ flies detected and avoided to eat the L-canavanine containing sucrose solution (black bars). On the contrary, Gr66a-Gal4/+;UAS-PTX/+ flies did not detect at all the L-canavanine as they ate the L-canavanine containing blue solution at the same level that the blue solution that did not contain L-canavanine (compare black and white bars in Fig. 5A, respectively). One hypothesis that could explain this result is that blocking Gαo47A function affects the development or the physiology of bitter-sensitive taste neurons. In order to exclude this hypothesis, we repeated the same experiment by using caffeine instead of L-canavanine. Caffeine is a potent repellent acting on bitter-sensitive taste neurons expressing GR66a [8]. As shown in Fig. 5A, Gr66a-Gal4/+;UAS-PTX/+ flies are strongly repelled by the presence of caffeine in the blue sucrose solution. This data strongly suggested that the impairment of Gαo47A function did not alter the development or the differentiation of the bitter-sensitive taste neurons in Gr66a-Gal4/+;UAS-PTX/+flies.

In order to confirm these data, we performed electrophysiological studies on the s6 sensilla of the proboscis, which is known to respond to bitter compounds [7]. As shown in Fig. 5B and 5C, Gr66a-Gal4 and UAS-PTX parental lines responded to 40 mM L-canavanine and 10 mM caffeine at approximately the same level. In contrast, no response was observed during L-canavanine stimulation on Gr66a-Gal4/+;UAS-PTX/+ flies. These data were confirmed on Gr66a-Gal4/+;UAS-RNAiGαo47A/+ flies, which had a strongly reduced response to L-canavanine (Fig. 5B and 5C), revealing that Gαo47A was required for L-canavanine perception. It is likely that the effect obtained by using PTX are stronger than the ones obtained with the RNAi-Gαo47A (or the Gαo^GDP^ construct, see Fig. 4) because the PTX-induced blockade of Gαo function is irreversible.

Importantly, we still detected a normal response during caffeine stimulation on Gr66a-Gal4/+;UAS-PTX/+ and Gr66a-Gal4/+;RNAiGαo47A/+ flies (Fig. 5B and 5C), confirming that their bitter-sensitive taste neurons were fully functional. To definitively exclude a role of Gαi65A in L-canavanine detection, we performed spike recordings on Gr66a-Gal4/+;UAS-RNAiGαi65A/+ flies and found no statistical significant differences compared to the Gr66a-Gal4 and UAS-RNAiGαi65A/+ control lines during L-canavanine or caffeine stimulation (Fig. S1B and S1C). Note that the decreased response observed between Gr66a-Gal4/+;UAS-RNAiGαi65A/+ and wild-type control flies during L-canavanine stimulation is very likely due to the UAS-RNAiGαi65A transgene insertion, which showed by itself a reduced response when crossed with wild-type control flies (Fig. S1B and S1C). Altogether, these behavioral and electrophysiological data show that PTX-induced Gαo47A inhibition and RNAi knock-down of Gαo47A strongly affect L-canavanine detection but have no effect on caffeine sensitivity.

## Discussion

The goal of this study was to explore the L-canavanine-induced signaling transduction pathway in bitter-sensitive GRNs of *Drosophila*. By using a multidisciplinary approach, we provided evidence that Gαo47A protein is required for L-canavanine detection.

Our study identified for the first time a *Drosophila* G protein subunit required for the detection of the toxic compound L-canavanine. Indeed, we demonstrated that rejection behavioral responses to L-canavanine (premature proboscis retraction and avoid eating) as well as electrophysiological response on proboscis sensilla known to respond to bitter compounds were dependent on active Gαo47A. These results are important since they are supporting our previous report showing that DmXR, a Gα_i/o_ coupled mGluR-like GPCR, is mediating the repellent effect of L-canavanine. We have no explanation for the recent results of Lee and collaborators reporting that flies missing DmXR displayed normal L-canavanine avoidance [22]. To gain further insight on L-canavanine associated signal transduction, we explored the involvement of heterotrimeric G proteins, which are crucial downstream effectors of GPCR signaling. Here, the inactivation of Gαo47A was obtained by different technical approaches, reducing a possible artifact. In addition, the behavioral and electrophysiological responses to caffeine were perfectly maintained in bitter-sensitive taste neurons in which Gαo47A was either down-regulated by using a RNAi-Gαo47A construct or blocked by using the pertussis toxin (PTX), excluding a general effect of the loss of Gαo47A function on signaling events involved in bitter sensing in those neurons.

The GR family is likely not belonging to the GPCR family of receptors because recent studies have revealed that insect GRs, like their related ORs, have an inverted topology relative to GPCRs with their N-terminus being intracellular and their C-terminus extracellular [16]. GRs are likely channels. This idea is reinforced by the recent study of Sato and collaborators that found that BmGr-9, a GR from *Bombyx mori*, constitutes a ligand-gated ion channel responding to D-fructose [41]. In *Drosophila*, GR33a was described as a co-receptor for most bitter compounds [9] but we found no evidence that this receptor was involved in L-canavanine detection (data not shown). However, Lee and collaborators reported that GR66a and GR8a are required for L-canavanine response [22]. Our experiments are not excluding that DmXR plus one or several GRs are required for a full response to L-canavanine. One hypothesis may be that L-canavanine binds to the GPCR DmXR that activates Gαo47A, to finally stimulate a complex of GRs containing at least GR66a and GR8a. Another hypothesis could be that L-canavanine acts on GR8a/GR66a and that a DmXR-linked metabotropic mechanism influences the GR-mediated signal transduction. What is the exact role of Gαo and its downstream effectors remains to be determined. A second messenger can be involved but a direct binding of Gαo47A and/or Gβ/γ subunits on GRs cannot be excluded [42]. A future challenge will be to identify the others players involved in L-canavanine detection.

Involvement of G proteins in bitter taste transduction was also found in other fly species. By using GDPβS, a competitive inhibitor of G-protein activation, Ouyang and collaborators found that strychnine and quinine detection in blowflies is dependent on a G protein signaling cascade [43]. While their approach did not allow them to unambiguously identify which subtype of G proteins is involved, their data suggest that the G protein-dependant signaling cascade is linked with the activation of phospholipase C and IP production, suggesting that the G protein involved there is a Gα_q_ subtype. Several studies have found an involvement of *Drosophila* G protein subunits in the detection of sugars. These G proteins include Gγ_1_ [44], Gα_s_ [45], Gα_q_ [46] and also Gα_o_ [47]. Interestingly, Bredendiek and collaborators found that Gα_o_ function is required in sugar-sensitive GRNs for the detection of sucrose, glucose, and fructose, but not for trehalose and maltose [47]. Altogether, this suggests that different sugars may activate different signaling pathways within sugar-sensitive GRNs. So, it seems that, at least in sugar and bitter-sensitive GRNs, distinct ligand may activate distinct signaling pathways leading to neuronal activation. It is important to note that in all these studies, G proteins were not “essential” for the transduction mechanisms as the response for tastants were never fully abolished when G protein function was impaired. In our study, we showed that blocking Gα_o_ function led to a very strong reduced response for L-canavanine, clearly indicating that Gα_o_ is a crucial downstream effector for L-canavanine detection by *Drosophila* bitter-sensitive GRNs.

Within the large family of GPCRs, DmXR belongs to the class C, which includes the metabotropic glutamate receptors, the GABA_B_ receptors, the calcium-sensing receptor as well as some taste and pheromone receptors. The mX receptors form a distinct group within the mGluRs subclass [21]. In vertebrates, there are eight mGluRs that can be distinguished in three groups based on their sequence homology and pharmacology. While all mGluRs are well known for their roles in the central nervous system [48], recent studies suggest that mGluR1 and mGluR4 subtypes are involved in the umami response [49], [50]. Umami taste, which is mostly elicited by L-glutamate, is also detected by heteromers of taste receptor type 1 (T1R1+T1R3) [51]. It is well known that the transduction cascade coupled to T1R1/T1R3 GPCRs relies on G proteins that will ultimately lead to the activation of the ion channel TRPM5 [52]. On the contrary, umami detection by mGluR1/4 seems to be independent of TRPM5 but the signaling cascade coupled to mGluR1/4 in taste buds remains to be elucidated [49]. It is interesting to note that these two mGluRs are coupled to different transduction pathways in heterologous systems: mGluR1 stimulates phospholipase C and phosphoinositide hydrolysis while mGluR4 inhibits adenylyl cyclase and cAMP production [33]. However, it could be that mGluR1 and mGluR4 form an heterodimer within taste buds and that this heterodimer has a unique coupling to G proteins. A future challenge will be to determine which G protein is required for umami detection in mice taste buds.

Most if not all bitter compounds previously used to study taste in insects, such as caffeine or quinine for example, lead to an inhibition of the proboscis extension reflex (PER) induced by sugar solution in contact with legs [38]. On the contrary, L-canavanine did not induce any inhibition of PER but rather a premature retraction of the proboscis (PPR), *i.e.* flies extend their proboscis but retract it immediately. This rejection behavior is sufficient to avoid L-canavanine ingestion. This difference of behavior may be explained by the fact that L-canavanine acts on a GPCR while other bitter compounds act on ligand-gated GRs, the metabotropic pathway being slower than the ionotropic pathway. This point is difficult to answer yet as it was never shown that bitter compounds, such as caffeine or quinine, act directly on GRs. In conclusion, future exciting studies will help to decipher the complex signaling pathways involved in taste transduction in *Drosophila*.

## Supporting Information

Figure S1 **RNAi knockdown of Gαi65A in bitter-sensitive taste neurons has no effect on L-canavanine and caffeine detection.** **A**) Two-choice feeding test experiments showing preference index for the blue solution of flies with different genotypes. Control indicated that no drug was added to the blue medium (white bars). Data obtained by using 30 mM L-canavanine or 10 mM caffeine in the blue medium are shown in grey and black bars, respectively. Compared to the Gr66a-Gal4/+ and UAS-RNAiGαi65A/+ (UAS-RNAiGi/+) control lines, Gr66a-Gal4/+;UAS-RNAiGαi65A/+ (Gr66a-Gal4/+;UAS-RNAiGi) flies did not show defect in L-canavanine aversion (ns p = 0.0542 and 0.6685, respectively). Note that aversion to caffeine was comparable for the three genotypes. Error bars indicate SEM. Statistical significant differences were analyzed by Unpaired Student's *t* test (ns: not significant). **B**–**C**) Electrophysiological recordings were performed from s6 sensilla on the proboscis of flies with different genotypes. The electrical activity of the taste neurons was recorded by capping taste sensillum with an electrode containing 1 mM KCl as an electrolyte and the stimulus (40 mM L-canavanine or 10 mM caffeine). **B**) Sample responses for 1 mM KCl, 40 mM L-canavanine (mentioned as L-cana) and 10 mM caffeine on Gr66a-Gal4 parental line, UAS-RNAiGαi65A/+ (UAS-RNAiGi/+) and Gr66a-Gal4/+;UAS-RNAiGαi65A/+(Gr66a-Gal4/+;UAS-RNAi/+) flies. **C**) No statistically significant differences were observed between Gr66a-Gal4/+;UAS-RNAiGαi65A/+ (Gr66a-Gal4/+;UAS-RNAi/+, black bars) flies and the Gr66a-Gal4 parental line (light grey bars) as well as the UAS-RNAiGαi65A/+ control flies (UAS-RNAiGi/+, dark grey bars) (p = 0.154 and 0.205 respectively). Note that a significant decrease of spike numbers is observed between UAS-RNAiGαi65A/+ flies and Gr66a-Gal4 parental line as well as the control. This likely due transgene insertion effect explains why Gr66a-Gal4/+;UAS-RNAi/+ flies showed a significant decrease of spike numbers during L-canavanine stimulation compared to wild-type control flies (white bars). Note that the response to 10 mM caffeine is not statistically different between all genotypes. The response was evaluated by counting the number of spikes elicited during the first second of the stimulation. N = 7–10 for each condition. Error bars indicate SEM. Asterisks indicate significant differences by Unpaired Student's *t* test (ns: not significant, * p<0.05, ** p<0.01, *** p<0.001).
(TIF)
