# Supplementary figures and images for: Gαo Is Required for L-Canavanine Detection in Drosophila
Source: PLoS One. 2013 May 6;8(5):e63484. doi: 10.1371/journal.pone.0063484 (PMC3646046; doi:10.1371/journal.pone.0063484)

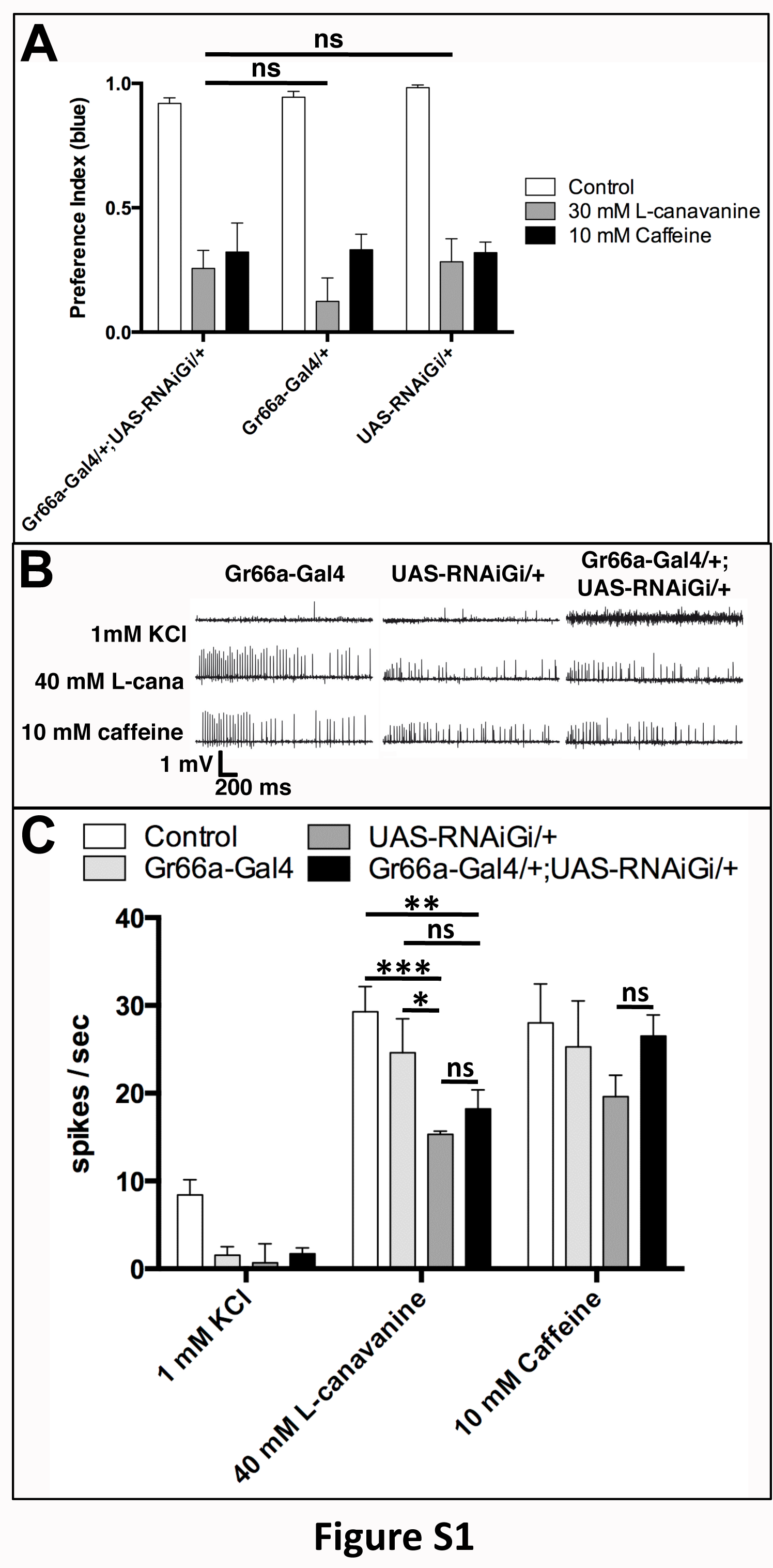

Supplement: Figure S1 — RNAi knockdown of Gαi65A in bitter-sensitive taste neurons has no effect on L-canavanine and caffeine detection. A) Two-choice feeding test experiments showing preference index for the blue solution of flies with different genotypes. Control indicated that no drug was added to the blue medium (white bars). Data obtained by using 30 mM L-canavanine or 10 mM caffeine in the blue medium are shown in grey and black bars, respectively. Compared to the Gr66a-Gal4/+ and UAS-RNAiGαi65A/+ (UAS-RNAiGi/+) control lines, Gr66a-Gal4/+;UAS-RNAiGαi65A/+ (Gr66a-Gal4/+;UAS-RNAiGi) flies did not show defect in L-canavanine aversion (ns p = 0.0542 and 0.6685, respectively). Note that aversion to caffeine was comparable for the three genotypes. Error bars indicate SEM. Statistical significant differences were analyzed by Unpaired Student's t test (ns: not significant). B–C) Electrophysiological recordings were performed from s6 sensilla on the proboscis of flies with different genotypes. The electrical activity of the taste neurons was recorded by capping taste sensillum with an electrode containing 1 mM KCl as an electrolyte and the stimulus (40 mM L-canavanine or 10 mM caffeine). B) Sample responses for 1 mM KCl, 40 mM L-canavanine (mentioned as L-cana) and 10 mM caffeine on Gr66a-Gal4 parental line, UAS-RNAiGαi65A/+ (UAS-RNAiGi/+) and Gr66a-Gal4/+;UAS-RNAiGαi65A/+(Gr66a-Gal4/+;UAS-RNAi/+) flies. C) No statistically significant differences were observed between Gr66a-Gal4/+;UAS-RNAiGαi65A/+ (Gr66a-Gal4/+;UAS-RNAi/+, black bars) flies and the Gr66a-Gal4 parental line (light grey bars) as well as the UAS-RNAiGαi65A/+ control flies (UAS-RNAiGi/+, dark grey bars) (p = 0.154 and 0.205 respectively). Note that a significant decrease of spike numbers is observed between UAS-RNAiGαi65A/+ flies and Gr66a-Gal4 parental line as well as the control. This likely due transgene insertion effect explains why Gr66a-Gal4/+;UAS-RNAi/+ flies showed a significant decrease of spike numbers during L-canava [file pone.0063484.s001.tif]
